# Lithium as an Alternative Option in Graves Thyrotoxicosis

**DOI:** 10.1155/2015/869343

**Published:** 2015-09-06

**Authors:** Ishita Prakash, Eric Sixtus Nylen, Sabyasachi Sen

**Affiliations:** Department of Medicine, Division of Endocrinology & Metabolism, Medical Faculty Associates, The George Washington University, Washington, DC, USA

## Abstract

A 67-year-old woman was admitted with signs and symptoms of Graves thyrotoxicosis. Biochemistry results were as follows: TSH was undetectable; FT4 was >6.99 ng/dL (0.7–1.8); FT3 was 18 pg/mL (3–5); TSI was 658% (0–139). Thyroid uptake and scan showed diffusely increased tracer uptake in the thyroid gland. The patient was started on methimazole 40 mg BID, but her LFTs elevated precipitously with features of fulminant hepatitis. Methimazole was determined to be the cause and was stopped. After weighing pros and cons, lithium was initiated to treat her persistent thyrotoxicosis. Lithium 300 mg was given daily with a goal to maintain between 0.4 and 0.6. High dose Hydrocortisone and propranolol were also administered concomitantly. Free thyroid hormone levels decreased and the patient reached a biochemical and clinical euthyroid state in about 8 days. Though definitive RAI was planned, the patient has been maintained on lithium for more than a month to control her hyperthyroidism. Trial removal of lithium results in reemergence of thyrotoxicosis within 24 hours. Patient was maintained on low dose lithium treatment with lithium level just below therapeutic range which was sufficient to maintain euthyroid state for more than a month. There were no signs of lithium toxicity within this time period. *Conclusion*. Lithium has a unique physiologic profile and can be used to treat thyrotoxicosis when thionamides cannot be used while awaiting elective radioablation. Lithium levels need to be monitored; however, levels even at subtherapeutic range may be sufficient to treat thyrotoxicosis.

## 1. Introduction

Lithium is an alkali metal used to treat bipolar disorder, which has been shown to have significant effects on thyroid function. Lithium carbonate has been used since 1948 to treat manic-depressive states [1], but it was not until the late 1960s and early 1970s that hypothyroidism and goiter were noted as side effects of long-term use of this medication [2, 3]. The rate of hypothyroidism varies from 0 to 47% [4], likely due to differences in definitions, study designs, and duration of lithium treatment. Lithium has been shown to significantly increase TSH as well as TRH-stimulated release of TSH [5]. This leads to hyperplasia of the thyroid gland and a nontender goiter formation. We describe a case of Graves' thyrotoxicosis that was difficult to control due to antithyroidal drug toxicity. In this case, the patient's hyperthyroid state was effectively controlled with lithium for almost two months.

## 2. Case Report

A 67-year-old female presented with seizures and fever to a different institution. The patient had an extensive medical history, including atypical hemolytic uremic syndrome, seizures, diabetes, and hypertension. Graves' disease was diagnosed during a prior hospitalization approximately 1.5 months earlier. Her TSH was undetectable, and TSI level was 658% (0–139%). Thyroid uptake and scan showed a moderately enlarged gland with diffusely increased tracer uptake at 43% at 24 hours (normal 8–32%). Methimazole and propranolol were started, at the other institution, but the patient developed fulminant hepatitis, which, after extensive workup, was determined to be due to the methimazole. Methimazole was withdrawn when the patient presented to our hospital. She was admitted with history of seizures. She was not tachycardic or hypertensive. She had a temperature of 103.9°F. The Burch-Wartofsky score was 35 [6] and the Glasgow Coma Scale score was 6. She was in normal sinus rhythm and showed no signs or symptoms of heart failure. However, TSH continued to be <0.015 mU/L (0.4–4.7), with elevated FT4 > 6.99 ng/L (0.7–1.8) and FT3 of 18 pg/mL [7–9]. She was started on 300 mg lithium daily which resulted in a serum level of 0.2 mmol/L (therapeutic 0.6–1.2 mmol/L). Other initial labs showed leukocytosis, mild anemia with occasional schistocytes, as well as a mild rise in AST and ALT (transaminitis), indirect bilirubinemia, and elevated lactate dehydrogenase level. The patient was being worked up for recurrent atypical hemolytic anemia and was deemed to be unsuitable to undergo thyroidectomy or to receive radioactive iodine ablation.

After a prolonged debate regarding choice of antithyroid medication we decided to initiate treatment with lithium. We initiated intravenous lithium therapy at 150 mg TID, and the dose was titrated to achieve serum lithium levels in the range of 0.4–0.6 mmol/L.

**Figure 1:**
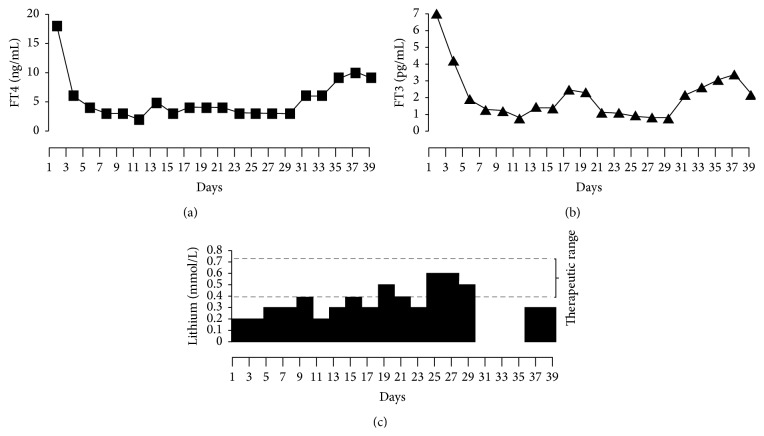
Correlation of free thyroid hormone levels and lithium levels over admission period. (a) shows the levels of free T4, followed by the levels of free T3 in (b), and corresponding lithium levels over the period of admission on (c). Note the low lithium level required to maintain* euthyroid* status over the entire course of hospital admission with no side effects.

Over the next 5 days TSH rose to 0.3, FT3 normalized, and FT4 decreased to 1.9 ng/L (Figure 1). We kept following active hormone levels, that is, FT3 and FT4, closely. The patient's mental status improved and she became verbal and coherent and was able to be transferred out of the intensive care unit. High dose glucocorticoids and propranolol, which were started initially, were weaned off and the patient gradually achieved biochemical and clinical euthyroid status. Free hormone levels completely normalized after 8 days of treatment at which time lithium level was 0.3 mmol/L (Figure 1). The lithium dose was stabilized at 300 mg total daily (split into twice daily dosing), to maintain levels between 0.4 and 0.6 mmol/L. The free hormone levels remained normal until 13 days after initial normalization of free hormone level with the lithium levels remaining between 0.4 and 0.6 mmol/L (Figure 1).

At this stage, the patient was moved out of the intensive care unit but remained admitted for observation.

On the 17th day, the patient experienced seizures (11 days after biochemical and clinical stabilization) and required readmission to intensive care unit with intubation. MRI scan showed features of acute cerebrovascular accident affecting the PCA and ACA territories. Patient remained clinically euthyroid.

We noted increase in FT4 and FT3 levels and at this stage the lithium levels were at 0.3 mmol/L (Figure 1). Lithium dosing was increased to 300 mg in the morning and 150 mg in the evening. On the new higher dose of lithium, the patient achieved biochemical euthyroid status within 4 days, and mental status improved. Patient remained biochemically euthyroid, with lithium level at 0.5–0.6 mmol/L (until 27 days after initial normalization).

On day 28 of admission, lithium was inadvertently stopped in the intensive care unit and patient did not receive lithium for the next 72 hours. Lithium levels reached undetectable levels in plasma and free hormone levels immediately rose again within 24 hours. Lithium was resumed at previous dosing (300 mg in the morning and 150 mg in the evening), with prompt improvement in biochemical thyrotoxicosis.

Over the next few weeks, the patient remained in euthyroid state. The patient was sent to a rehabilitation facility to recover appropriately. While at the center the patient was maintained on twice daily lithium. Her TSH and free T4 remained satisfactory even on a small dose of lithium (at subtherapeutic serum level of lithium) without any fluctuations of her electrolytes and liver enzymes.

## 3. Discussion

We have described a patient with thyrotoxicosis who developed liver toxicity on thionamide therapy and was subsequently successfully treated with a prolonged low dose of lithium, without any side effects.

Hepatotoxicity due to methimazole tends to be more of a cholestatic process, rather than hepatocellular injury and allergic hepatitis seen with propylthiouracil toxicity [7]. Once hepatotoxicity is established, the medication must be stopped urgently. Generally, an alternative thionamide is not recommended due to the reported cross-reactivity between formulations, which is noted to be as high as 50% [10]. In cases of resistant hyperthyroidism, definitive treatment with radioactive iodine or total thyroidectomy is considered as soon as possible [11]. Plasmapheresis use has been documented in limited case reports of life-threatening, resistant thyrotoxicosis since the 1970s; however there are no randomized trials or guidelines for its use [12].

Other agents such as beta blockers, steroids, iodine, cholestyramine, and lithium have traditionally been used as a temporizing bridge to definitive therapy.

On initial presentation at our center, in our patient, adequate dose of propranolol was used (40 mg three times daily) titrated to heart rate along with 4 mg dexamethasone. Iodine therapy was considered and indeed the initial use of lithium by the outside institution showed hyperthyroid efficacy. However patient on initial presentation was very unstable and we decided to use lithium so as not to prevent the radioiodine option and also the patient had a diagnostic whole body CT scan with contrast few days prior to her admission, which could make further iodine loading redundant. Moreover, our long-term management plan was to utilize the radioiodine concentration ability of lithium to achieve a permanent cure of Graves' disease.

In that scenario lithium appears to be a good alternative. It is highly concentrated in the thyroid follicular cells. It has been shown to inhibit iodine uptake, interfere with tyrosine iodination, change the thyroglobulin structure, and interfere with iodotyrosine synthesis [13]. Lithium is also known to interfere with the sodium-iodide symporter and block iodine uptake into the follicular cell, reducing the substrate needed to create thyroid hormone. Lithium blocks thyroid hormone release from thyroglobulin, which in turn inhibits adenylate cyclase and prevents thyroid stimulating hormone or thyroid stimulating antibody from stimulating the cell via the thyroid hormone receptor [14]. Lastly, lithium inhibits deiodinases in the periphery.

Lithium has been shown to increase the retention of radioactive iodine (RAI) in the thyroid of patients with Graves' thyrotoxicosis [15], in turn, leading to improvement of the efficacy of this therapy. Lithium given before or concomitantly with RAI has been shown to provide more immediate control of hyperthyroidism, by decreasing the release of preformed thyroid hormone, without decreasing the uptake of the RAI [16]. This effect is reminiscent of the Wolff-Chaikoff effect, where increased iodine content inside the follicular cell blocks release of hormone. This effect, much like the effect of iodine, is transient. Older literature notes the similarity of lithium to iodine and recommends its use only for short-term, rapid suppression [17]. However, our case highlights the fact that lithium has several other mechanisms of action that contribute to long-term control of thyrotoxicosis.

There are few cases in the literature that describe use of lithium as a stand-alone drug to treat thyrotoxicosis, without plan for definitive treatment. In one such study, lithium was the only drug used to treat 11 patients with Graves' disease who relapsed after treatment with conventional antithyroid medications, radioiodine treatment, or surgery [18]. Patients were given lithium for 6 months at a dose ranging from 800 to 1200 mg daily, with levels in the blood ranging from 0.5 to 1.5 mmol/L. Eight of the patients studied became clinically euthyroid 2 weeks after starting lithium, with thyroid hormone levels decreasing by a mean of 35%. Another 3 patients took 4–6 weeks to achieve euthyroid state. The important finding was that euthyroid state was maintained after 6 months, and seven patients relapsed into thyrotoxicosis after lithium was stopped for 1–4 weeks.

Lithium reaches peak plasma concentration in 1-2 hours for the immediate release formulation and 4-5 hours for sustained release formulation. A steady state in the body is usually achieved after 4 days [14]. Apart from the effects on the thyroid, reported adverse effects include a spectrum of central nervous system, cardiovascular, and renal side effects. These include confusion, coma, seizures, ventricular irritability, sinus node dysfunction, sinoatrial block, nephrogenic diabetes insipidus, electrolyte imbalance, hypercalcemia, and hypermagnesemia. Studies have shown that lithium doses of 600 mg−1000 mg daily (300 mg every 8 hours), as well as lithium blood levels of 0.6–1.2 mmol/L, are best to control thyrotoxicosis. To avoid toxicity, it is best if serum lithium levels are maintained < 1.0 mmol/L, around 0.5 mmol/L. [14].

Our case illustrates that a low therapeutic level of lithium even around 0.2 mmol/L is sufficient to suppress thyroid overactivity without causing side effects. Lithium at a low dose appears to be an effective antithyroid medication even for a few months.

## 4. Conclusion

Lithium can be used to treat thyrotoxicosis when other more commonly used therapeutic options are not available, and therapy duration beyond a few weeks is a possibility. Moreover, as shown in this case, lithium doses in the low, subtherapeutic range can still be effective in controlling thyrotoxicosis even over several weeks.
